# Surgical Phase of the Tonsil-Adenoid Clinic

**Published:** 1920-11

**Authors:** Edwin S. Ingersoll


### SURGICAL PHASE OF THE TONSIL-ADENOID CLINIC

BY EDWIN S. INGERSOLL, M.D.

The plan was to provide for forty operations a day, five days a week, over a period of seven weeks. With a normal capacity of twelve cases, three times a week, the ordinary equipment of the surgical department was far from adequate and necessitated a complete reorganization and the use of other parts of the building not hitherto used for surgical purposes. Also the increase in the number of cases operated on made necessary an entirely new reorganization of the surgical, medical and nursing staffs.

At first it was planned to have four operators work each day, in two shifts, as but two tables could be used at a time; but that plan was soon abandoned, as it was found more satisfactory to have two men take care of each day's work. Dr. Henry W. Edwards and Dr. Warren Wooden did by far the greatest amount of the operating, each working at least four days a week, and sometimes five. Dr. Robert L. Ideson operated one day each week and Dr. Ralph Honiss was available when it was found necessary to call upon him.

Dr. Stearns S. Bullen was in charge of the anaesthesia, assisted by Dr. E. W. Phillips and Dr. John Fowler. These anaesthetists were sufficient to keep the operators supplied with anaesthetized cases. Ether was used in all cases.

Two resident physicians, Dr. Richard Leonardo and Dr. Walter Edwards were on duty, both being in the dispensary during the day, and one of them at night. Dr. Albert D. Kaiser was in charge of the medical supervision.

Eight graduate nurses were employed, five for day duty and three at night.

At the time of admission special note was made of any child having a temperature above 99.2, and unless the temperature the following morning was normal, the child was sent home without operation. A few cases of incipient acute tonsilitis, bronchitis and intestinal upset were discovered during the routine examination the day before operation, and these cases were invariably held up for observation and parents instructed to apply for a later appointment after the physical condition had become normal.

Two tables were used in the operating room. Both tables were supplied with instruments and materials from one main supply table in charge of a scrubbed nurse. The nurse handled only clean instruments and supplies at each session, the used instruments being collected by another nurse, cleaned and sterilized before being again placed with the sterile supply.

The technique used in the large majority of cases was that of Beck, using the fenestrated snare. There were some cases of secondary removal when dissection was necessary, but these were comparatively few in number. The average time of all operations was six minutes, twenty-one seconds.

During the first two weeks of the clinic there were three or four cases each day which bled somewhat more than normal sometime during the day or night following operation. None of these cases were at all alarming, except one hemophiliac who required transfusion. This case subsequently made a perfect recovery. The cards of hemophiliacs were marked in red ink.

Beginning with the third week of the clinic, at the suggestion of Dr. A. C. Magruder, of Colorado Springs, Colo., a sponge moistened with a 40 per cent. acacia solution of turpentine was applied after the tonsil was removed. After this, the number of cases that bled was greatly reduced and the amount of vomiting during the first three post-operative hours was markedly less.

A significant feature of the clinic was the large proportion of children examined who showed imperative need of removal or tonsils and adenoids. One explanation of this is probably that all of the children presented for examination had previously been told by school nurse or family practitioner that attention was necessary. There was not a case operated on during the entire clinic where the operation was not indicated either from the standpoint of respiratory obstruction, or of infection to an extent detrimental to the child's health.

It is, of course, too early to determine the beneficial results which are confidently expected to ensue, and there is material for a large amount of further investigation. In these respects, the clinic should prove of great scientific value. Probably never before has there been such a large number of operations of this nature in the same space of time and under conditions permitting the cases to be kept under such close observation. It is therefore to be expected that two or three years hence it will be possible to prepare a report that will establish more exactly and conclusively than any existing data, the true effect of the removal of tonsils and adenoids in children under sixteen years of age.

---

